# Immunomodulatory and Allergenic Properties of Antimicrobial Peptides

**DOI:** 10.3390/ijms23052499

**Published:** 2022-02-24

**Authors:** Svetlana V. Guryanova, Tatiana V. Ovchinnikova

**Affiliations:** 1 M.M. Shemyakin and Yu.A. Ovchinnikov Institute of Bioorganic Chemistry RAS, 117997 Moscow, Russia; ovch@ibch.ru; 2 Medical Institute, Peoples’ Friendship University of Russia, 117198 Moscow, Russia; 3 Phystech School of Biological and Medical Physics, Moscow Institute of Physics and Technology, 141701 Dolgoprudny, Russia; 4 Department of Biotechnology, I.M. Sechenov First Moscow State Medical University, 119991 Moscow, Russia

**Keywords:** antimicrobial peptides, defensins, cathelicidins, innate immune system, immunomodulatory activity, allergenic properties

## Abstract

With the growing problem of the emergence of antibiotic-resistant bacteria, the search for alternative ways to combat bacterial infections is extremely urgent. While analyzing the effect of antimicrobial peptides (AMPs) on immunocompetent cells, their effect on all parts of the immune system, and on humoral and cellular immunity, is revealed. AMPs have direct effects on neutrophils, monocytes, dendritic cells, T-lymphocytes, and mast cells, participating in innate immunity. They act on B-lymphocytes indirectly, enhancing the induction of antigen-specific immunity, which ultimately leads to the activation of adaptive immunity. The adjuvant activity of AMPs in relation to bacterial and viral antigens was the reason for their inclusion in vaccines and made it possible to formulate the concept of a “defensin vaccine” as an innovative basis for constructing vaccines. The immunomodulatory function of AMPs involves their influence on cells in the nearest microenvironment, recruitment and activation of other cells, supporting the response to pathogenic microorganisms and completing the inflammatory process, thus exhibiting a systemic effect. For the successful use of AMPs in medical practice, it is necessary to study their immunomodulatory activity in detail, taking into account their pleiotropy. The degree of maturity of the immune system and microenvironment can contribute to the prevention of complications and increase the effectiveness of therapy, since AMPs can suppress inflammation in some circumstances, but aggravate the response and damage of organism in others. It should also be taken into account that the real functions of one or another AMP depend on the types of total regulatory effects on the target cell, and not only on properties of an individual peptide. A wide spectrum of biological activity, including direct effects on pathogens, inactivation of bacterial toxins and influence on immunocompetent cells, has attracted the attention of researchers, however, the cytostatic activity of AMPs against normal cells, as well as their allergenic properties and low stability to host proteases, are serious limitations for the medical use of AMPs. In this connection, the tasks of searching for compounds that selectively affect the target and development of an appropriate method of application become critically important. The scope of this review is to summarize the current concepts and newest advances in research of the immunomodulatory activity of natural and synthetic AMPs, and to examine the prospects and limitations of their medical use.

## 1. Introduction

Antimicrobial peptides (AMPs) are an ancient and widespread class of compounds widely-spread from prokaryotes to eukaryotes and aimed at host defense from pathogen penetration [1,2,3].

AMPs contain from 5 to 100 amino acid residues, including basic (arginine, lysine, histidine) and hydrophobic (often more than 50%) residues. The classic cationic antimicrobial peptides are defensins and cathelicidins.

The history of AMPs began in 1939 when a peptide in soil from Bacillus bacteria showing a protective effect against pneumococcal infection in mice was discovered and called gramicidin [4,5]. AMPs of animal origin, exhibiting lytic activity against Gram-negative bacteria, were first described in 1956: they were found in polymorphonuclear leukocytes of rabbit peritoneal exudate. In 1963, Zeya and Spitznagel isolated AMPs with a significant proportion of arginine from granules of rabbit and guinea pig neutrophils [5,6,7]. Afterwards, they were called defensins.

In 1981, Boman and colleagues isolated AMPs from the hemolymph of pupae of the butterfly *Hyalophora cecropia* and named them cecropins A and B [8,9,10]. The discovered peptides shed light on ways to protect insects against infection despite they lack the immunocompetent cells of adaptive immunity that provide protection for invertebrates. More than 250 AMPs from insects were isolated over time [11], many of which demonstrated not only lytic properties and the ability to kill Gram-positive and Gram-negative bacteria, but also prevented the formation of biofilms and the emergence of antibiotic-resistant strains. For example, cecropin A, could destroy the uropathogenic strain of *Escherichia coli* [12].

In 1985, Ganz, Lehrer and their colleagues for the first time characterized and determined the structure of AMPs from azurophilic granules of human neutrophils, designated as HNP1, HNP2, and HNP3 (Human Neutrophils Peptides) and named “defensins”. It was found that a mixture of three defensins HNP 1–3 at a concentration of 50 μg/mL had in vitro lytic activity against *Staphylococcus aureus*, *Escherichia coli* and *Pseudomonas aeruginosa*. Comparative analysis showed that HNP-1 and HNP-2 were more active than HNP-3 against most of the tested microbes [13].

Interest in AMPs increased significantly in 1986 when Zasloff discovered magainins, the peptides of mucous membranes of the clawed frog *Xenopus laevis*. These peptides are active not only against bacteria, but also against protozoa [14].

In 1991, a significant degree of homology between peptides of human, rabbit, guinea pig, and rat neutrophils was established and their decisive role in the implementation of the first line of defense against infections was determined [15].

In humans, AMPs were found not only in neutrophils, but also in natural killer cells, macrophages, T-lymphocytes, epithelial cells of the mucous membranes and skin [16,17,18], adrenal chromaffin cells [19] and testes [20]. Moreover, they possessed not only antibacterial [16,17,18], but also antiprotozoal [21,22,23], antifungal [23,24] and antiviral activity [25,26], and were shown to be involved in neuro-endocrine regulation [19,20].

**Figure 1:**
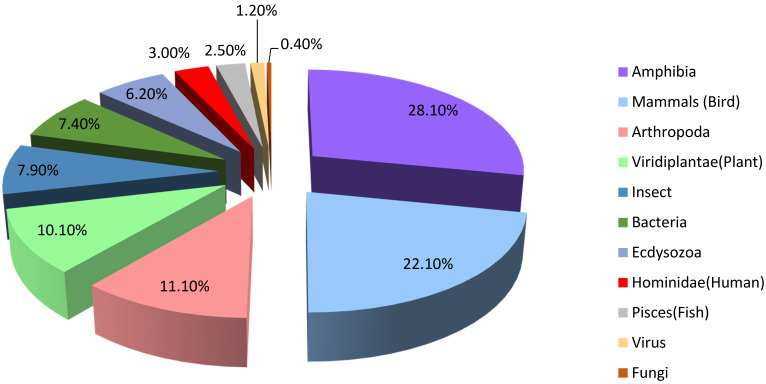
Natural sources of AMPs.

To date, the structures of 26,447 AMPs are known and 3444 of them are molecularly characterized with defined tertiary structure (Figure 1) [27].

Systematization of AMPs can be based on sources and methods of production of amino acid sequences and secondary structures (alpha-helical, beta-folded, beta-hairpin, cyclic, linear) [28]. Most AMPs acquire the aforementioned configurations when interacting with biological membranes. In this case, hydrophilic amino acid residues are located along one side, and hydrophobic amino acid residues are located along the opposite side of the molecule. Amphipathicity of AMPs allows them to be incorporated into the lipid bilayer of the membrane [28]. While membrane surfaces of eukaryotic cells are electrically almost neutral, bacterial surfaces are negatively charged due to the presence of phospholipids, teichoic acids, and lipopolysaccharides (LPS) and bind to positively charged AMPs via electrostatic interactions [29]. Such an affinity of cationic AMPs for bacterial membranes, but not for the membranes of host cells, ensures their specificity [30,31].

## 2. Antimicrobial Activity

The main function of AMPs is bactericidal or bacteriostatic action. Antimicrobial activity of AMPs is realized via direct interaction with bacterial cell walls based on electrostatic forces. Cationic AMPs interact with the negatively charged surface of the outer membrane of the bacterium and either neutralize its charge, create pores and penetrate the outer membrane, or bind to LPS and destroy the membrane. As soon as the peptides pass through the outer bacterial membrane, they bind to phospholipids of the negatively charged surface of the cytoplasmic membrane of the bacterium and cause fatal changes in the membrane structure or create transmembrane channels. As the result, the integrity of the bacterial cell is disturbed and its death occurs [32].

In most cases, the lytic activity of AMPs manifests itself in a destructive effect on bacterial membranes, but, in addition, a number of AMPs have the ability to affect the cytoplasmic targets of bacteria [33], inhibiting the synthesis and metabolism of nucleic acids [34,35,36], ATP [37,38], metalloproteinases [39,40,41], protein biosynthesis and folding [42,43,44], cell division [45,46,47], cell wall biosynthesis [48,49,50] and lipopolysaccharide biosynthesis [51,52,53]. Activity of AMPs can decrease with increasing acidity and ionic strength of the solution up to complete inhibition in solutions with pH 5.8 and in 0.14 M NaCl [54].

AMPs are produced not only by multicellular organisms to prevent infection, but also by unicellular organisms. Bacteria and protozoa produce AMPs with the aim of conquering habitats and influencing other representatives of the bacterial community. For example, microcins from Gram-negative bacteria *Enterobacterales* reduce the number of *E. coli* cells and increase the population of *Bifidobacterium* and *Lactobacillus* in the cecum [55], while AMP from the pathogenic representative of the Protozoan *Entamoeba histolytica* has demonstrated lytic properties against both Gram-positive and Gram-negative bacteria [56]. In addition to direct action on bacteria, AMPs realize their activity through interaction with pathogenic factors of bacteria, for example, with LPS, causing its subsequent inactivation [57,58,59]. The ability of AMPs to inactivate LPS was the basis for a large-scale study of their ability to prevent septic complications in the hope of further use in surgical interventions [60].

The potentiating effect of AMPs was demonstrated when using them in combination with antibiotics. The AMP arenicin-1 from the lugworm *Arenicola marina* is able to significantly facilitate the access of antibiotics to the intracellular target by destabilizing the membrane. The synergism of AMPs with antibiotics can reduce the dosage of antibiotics while maintaining their activity, which may significantly reduce the drug load on the human organism [61].

It should be noted that commensal and opportunistic pathogens show different sensitivity to AMPs. In particular, the resistance of the *Lactobacillus delbrueckii subsp. lactis* (CIDCA 133) to human alpha- and beta- defensins [62] and to AMPs of anuran amphibian (Aurein 1.2, Citropin 1.1 and Maculatin 1.1) [63] was observed, and the defensin activity at a concentration of 0.1–10 μg/mL was maximum in distilled water and inhibited in phosphate buffered saline (PBS). It turned out that resistance was associated with the structure of the peptidoglycan and the bacterial membrane of the CIDCA 133 strain as distinct from the CIeDCA 331 strain. The data obtained indicate that the CIDCA 133 strain of the *Lactobacillus delbrueckii subsp. lactis* is well adapted to the innate immunity effectors of both mammals and amphibians. This indicates conservative mechanisms of interaction of commensal microorganisms with key components of the host innate immune system.

Adaptation of microorganisms to AMPs can be expressed not only in changes in the structure of peptidoglycan and bacterial membranes, but also in the production of enzymes that destroy defensins of the macroorganism. For example, *Staphylococcus aureus* and *Streptococcus pyogenes* produce proteinases that destroy human defensins, thus ensuring the colonization resistance of bacteria [64,65,66].

The peculiarities of the response of various microorganisms to human AMPs should be studied in detail in order to develop a therapy strategy associated with infectious pathologies.

Many AMPs have antiviral activity in addition to antibacterial activity [67,68]. The virus neutralizing effect of AMPs is expressed both in direct interaction with the virus capsid and indirectly by activating the host antiviral immunity [69]. In the case of direct contact of AMP with the virus, the virus capsid may be destroyed [70,71,72] or the virus may be prevented from binding to the target cell receptor [73]. In particular, the possibility of hBD-2 binding to the RBD motif of the SARS-CoV-2 receptor responsible for interaction with the ACE2 receptor was found by molecular dynamics modeling, and was confirmed by biochemical methods. In addition, the dissociation constant of 300 nM was determined. In this case, the retention of the disulfide bond was the determinal factor. hBD-2 binds RBD and competitively inhibits RBD binding to ACE2 and inhibits virus entry into cells expressing ACE2 [74]. Cathelicidin LL-37 also directly interacts with the envelope of the SARS-CoV-2 virus and destroys it, which leads to virus inactivation [75,76]. The cyclic theta-defensin of the monkey rhesus *Macaca mulatta* and of baboons protects laboratory animals from severe acute respiratory syndrome coronavirus infection SARS-CoV [77]. The amphibian-derived peptides caerin 1.1, caerin 1.9, and maculatin 1.1 completely inhibited HIV infection of T cells within minutes of exposure to the virus at concentrations non-toxic to target cells [72]. Cyanovirin-N derived from cyanobacteria and binding the HIV envelope glycoprotein gp120 has a protective effect against HIV infection [78,79]. HNP1 directly inhibits the activity of HIV-1, HSV-1, HSV-2, VSV, influenza virus, CMV, adenovirus and papillomavirus [80]. Gray short-tailed opossum cathelicidins not only lyse Gram-positive and Gram-negative bacteria, but also inhibit the replication of West Nile virus (WNV) [81]. The cationic antimicrobial peptide caerin 1.1 from amphibians is considered as a promising drug in animal husbandry due to its lytic activity against porcine epidemic diarrhea virus (PEDV) [71], causing significant loss of livestock [82]. AMPs with a wide range of biological activities, including neurotoxic, anticoagulant, antidiabetic and antifreeze, have been isolated from the tissues of many marine invertebrates [83] [84,85].

The discovered ability of AMPs to prevent the appearance of biofilms [12,80,86], and their antiviral [67,80,81], antifungal [87,88,89], antiprotozoal [90], and antitumor [91,92,93,94] effects, as well as their immunomodulatory activity, serves as a serious basis for the development of modern medicines [95,96].

## 3. Immunomodulatory Activity of AMPs

AMPs in humans are represented by three main families: defensins, cathelicidins, histatins. Defensins, depending on the type of disulfide bond arrangement, are divided into alpha- and beta-defensins. Alpha- and beta-defensins are constitutively produced by neutrophils, lymphocytes, and epithelial cells of the skin and mucous membranes [97].

Human alpha defensins 1–4 are also called human neutrophil peptides 1–4 (HNPs), since they are most often expressed by neutrophils and account for up to 50% of the total protein content in these cells [98,99]. These peptides play a key role in local and systemic innate immunity, since they, together with other proteins such as lysozyme, proteases and RNases, etc., are involved in the destruction of bacterial pathogens [100]. The concentration of HNP 1-3 in human blood plasma is normally of 254.8 ± 7.1 pg/μL, and it can increase by 4.2 times in bacterial infection and 3.2 times in non-bacterial infection [101]. Human alpha defensins 5 and 6 are produced and secreted mainly by Paneth cells in the small intestine [102], as well as by epithelial cells of the female [103,104] and male genital organs [105].

Beta-defensins are expressed by epithelial cells of the skin and mucous membranes [106,107,108]. Six types of human beta-defensins (designated as hBD-1-6) have been isolated. The hBD-1, hBD-2 and hBD-3 defensins are well characterized in terms of expression levels and antimicrobial activity [109,110,111]. hBD-1 is constitutively expressed by epithelial cells, while hBD-2 and -3 are expressed by epithelial cells after stimulation with pro-inflammatory cytokines as well as with microorganisms. The antimicrobial activity of hBD-1 and hBD-2 decreases with increasing salt concentration, while hBD-3 retains its bactericidal effect even at high salt concentrations [112,113,114].

Defensin secretion is induced both by whole bacteria and by their components, for example, by LPS, lipoteichoic acid, lipid A, and muramyl peptides [115,116,117]. After it was shown that CpG oligodeoxynucleotides also induce the secretion of defensins, it became obvious that stimulation of the TLR or NLR receptors of the innate response is the signal for the release of defensins by cells [118,119,120]. AMP inducers of both endogenous and exogenous origin are of special interest due to their possible use as priority drugs that target AMP-producing cells. The induction of endogenous antimicrobial peptide expression is a new concept for the treatment of infections in humans, poultry and farm animals [121,122,123].

It was demonstrated that the gut microbiota-derived metabolites, more specifically short-chain fatty acids (SCFAs), promote the pancreatic production of cathelicidin, and the alteration in the gut microbiota explains the defective production of cathelicidin in NOD mice [124]. The short-chain butyric fatty acid and the diterpenoid forskolin were shown to induce the expression of the chicken AMP genes *AvBD2*, *AvBD6*, and *AvBD7* related to beta-defensins and cathelicidin *CATH2* both in vitro and in vivo [125]. In addition, vitamin D in the presence of bacterial pathogens is a direct inducer of the expression of genes encoding antimicrobial peptides, in particular cathelicidin [126]. This proves that AMPs are key factors of innate immune responses to bacterial infection and may act as signaling molecules participating in immune system regulation.

The level of expression of defensins is also influenced by tissue damage and endogenous factors. When tissue is damaged, the expression of hBD-2 increases, while insulin-like growth factor-1 (IGF-1) and transforming growth factor-alpha (TGF-alpha) suppress hBD-2 expression [127]. Rapid release of defensins can induce IFN-γ [128] and IL-13 [129].

The influence of defensins on certain representatives of the microbial community was investigated, and their influence on the microbiome and, as the result, on the homeostasis of the macroorganism was established. Due to the key role of defensins in the regulation and management of the human microbiome throughout development [130], the use of AMPs in medicine seems to be a worthy alternative to antibiotics.

Representatives of the cathelicidin family have been found in phagocyte granules, as well as in cells of various barrier epithelia. Unlike defensins, cathelicidins are characterized by a broad structural variety. However, these structurally diversified peptides are combined into one family due to the fact that all of them are formed from precursor molecules [131], including a highly conserved region (*cathelin domain*) homologous to the cathelin protein from porcine leukocytes. The first representatives of this class were the BtBac5 peptides [132] and dodecapeptide [133] isolated from bovine neutrophils. In humans, only one cathelicidin (hCAP18/LL-37) has been found in various types of epithelial cells [134]. The synthesis of this peptide is stimulated by infectious and inflammatory processes. Cathelicidins display a broad spectrum of antimicrobial activity, and some of them are able to bind and neutralize endotoxins. The activity of these peptides and their localization in neutrophils and mucosal epithelia allow one to consider them as the key effector molecules of the mammalian innate immunity system.

When introducing AMPs into medical practice, it is important to study their effect on the human immune system, in particular, on humoral and cellular immunity.

### 3.1. The Effect of AMP on Humoral Immunity

The effect of AMPs on humoral immunity was assessed by their ability to stimulate the production of antibodies, cytokines, and complement activation.

#### 3.1.1. Adjuvant Activity of AMPs

AMPs, as components of innate immunity, take part in triggering an antigen-specific immune response, promote intercellular cooperation and increase the production of antibodies. It was shown that intranasal administration of ovalbumin (OVA) together with defensins HNP1–3 to mice increased the production of specific IgG and IgM, but not IgA. The authors of the study concluded that defensins enhance the systemic immune response, but not the mucosal one [135].

Intraperitoneal administration of defensins HNP 1–3 to mice significantly increased the production of KLH-specific antibodies IgG1, IgG2a, and IgG2b after 14 days from immunization. Defensins also significantly increased the production of antibodies to the antigen of a syngeneic tumor and increased the resistance of animals to a transplantable tumor [136]. These results indicate that defensins act as potent immune adjuvants, enhancing the production of antigen-specific immunoglobulins.

The adjuvant activity of defensins is used in the development of vaccines against viral and bacterial infections. In particular, the mycobacterium *Mycobacteroides*, which is pathogenic to humans and frequently causes postoperative infectious complications, and is also found in patients with soft tissue infections, is very resistant to conventional antimicrobial drugs. The addition of hBD-2 as an adjuvant to the vaccine against *Mycobacteroides* has increased the effectiveness of therapy [137]. Defensin hBD-2 enhances the antigen-specific immune response not only against bacterial, but also toward viral antigens. The introduction of hBD-2 defensin into the vaccine increases the immunogenicity of vaccines against hepatitis B [138] and hepatitis C, both in free [139] and conjugated with a polypeptide form [140]. hBD-2 is being introduced as an adjuvant in MERS-CoV vaccines under development [141,142,143].

The adjuvant effect of human defensins, manifested against bacterial and viral infections, made it possible to formulate the concept of a “defensin vaccine” as a conceptual basis for constructing vaccines [144].

The effectiveness of the adjuvant impact of defensins increases by several times when administered together with hBD-2 or hBD-3 and with CpG nucleotides. Intraperitoneal immunization of mice with hBD2,3/CpG complexes increased the humoral response to OVA in comparison with only OVA/hBD3 or OVA/CPG by 5 and 10 times, respectively [145].

In the current epidemiological situation, researchers from different countries are using defensins to create vaccines against SARS-CoV-2. Approaches are being developed for conjugating defensins with T- and B-cell epitopes in vaccines against SARS-CoV-2. It has been shown that the binding of three structural polypeptides (spike, membrane and nucleocapsid (SMN)) with hBD-2 and hBD-3 at the N- and C-termini, respectively, increases the immunogenicity of the vaccine in the absence of an allergenic effect [146,147,148].

#### 3.1.2. The Effect of AMPs on the Cytokine and Chemokine Production

Constitutively synthesized AMPs maintain a balance of immune homeostasis not only through direct action on the pathogen and its elimination, but also via activation of the production of cytokines and chemokines that attract immunocompetent cells to the pathogen invasion zone. Thus, a multilevel protection of the body against infection is realized.

In a comprehensive study of the effect of hBD-1, hBD-2, and hBD-3 on the production of cytokines by peripheral blood cells (PBMC), the selective activity of defensins was shown [149]. hBD-1 increased the production of IL-6, IL-8, IL-10, MCP-1 (Monocyte Chemoattractant Protein 1, CCL2), and EGF (Epidermal Growth Factor) on the first day. IGFBP-3 (Insulin-like Growth Factor-Binding Protein 3) increased on the first day and decreased after 6 days. hBD-1 significantly lowered IL-5 and had no effect on IL-1-β, IL-16, and MCP-2. hBD-2 dose-dependently stimulated the induction of cytokines IL-1-β, IL-6, IL-8, IL-10, ENA-78 (CXCL5), IGFBP-3 (Insulin-like growth factor-binding protein 3), EGF (Epidermal growth factor), and HGF (Hepatocyte growth factor). The maximum activity in relation to IL-6 and IL-10 production was observed after 8 h, and that of IL-8 was observed after 18 h. hBD-3 exhibited the least activity of the three defensins, increasing only IGFBP-3 and MCP-1 slightly, while decreasing the level of IL-10 and HGF (Hepatocyte Growth Factor). Interestingly, some cytokines such as IL-8 and MCP-1 were activated by all three defensins, while IL-16 was activated by none of the tested defensins. It is noteworthy that each defensin induced a unique set of cytokines. A multidirectional action of defensins with respect to IL-10 was found: hBD-1 and hBD-2 activated its synthesis, while hBD-2 inhibited it.

In experimental studies of the effect of hBD-1 on human bronchial epithelial cells, a dose-dependent increase in IL-8 and IL-1 was observed [150]. While investigating human keratinocytes, it was shown that at a concentration of 5 to 8 μg/mL, hBD-2, hBD-3, and hBD-4 but not hBD-1 had a stimulating effect, which led to an increase in the production of IL-6, IL-10, MCP-1, and MIP-3. In this case, the cytotoxic effect of defensins was manifested at a dose of 50 µg/mL [151,152].

hBD-2 and hBD-3, when co-administered with CPG, increased the IFN-α synthesis by human plasmacytoid dendritic cells and induced inflammation. Intravenous (I.v) administration of hBD3/CpG complexes to mice induced the production of proinflammatory cytokines such as IL-12, IFN-γ, IL-6, IFN-α, and IL-10 in blood serum [145].

Alpha defensins 1–3 also increased the ex vivo production of IFN-γ in KLH-activated spleen cell supernatants from mice [136].

Intranasal immunization of mice with ovalbumin (OVA) with the subsequent administration of human alpha defensins 1–3 increased the production of IFN-γ, IL-5, IL-6, and IL-10 compared to control groups immunized with ovalbumin [135].

When studying the effect of alpha-defensins HNP1–3 on human lung epithelial A249 cells with regards to the production of pro-inflammatory and anti-inflammatory cytokines IL-1 β, IL-2, IL-4, IL-6, IL-8, IL-10, IL-12, TNF-α, IFN-γ and GM-CSF, a dose-dependent ability of HNP1–3 to induce IL-8 production was found. It is important to note that the HNP-induced IL-8 release was observed even at very low doses (3 μg/mL) of alpha-defensins [98].

Human AMP cathelicidin LL-37 induces the production of cytokines IL-6, IL-8 and IL-10, as well as of CC-chemokine ligand 2 (CCL2) [153] and can act synergistically with IL-1β to increase the production of cytokines IL-6, IL- 8 and IL-10, as well as the chemokine CCL2. Cathelicidin LL-37 also increases the synthesis and release of alfa-defensins, forming a positive feedback loop that enhances the inflammatory process [154]. Similarly, hBD-1 and HNP-1, acting on dendritic cells, also enhance their own expression [155].

In some cases, the induction of pro-inflammatory cytokines may be undesirable, and therefore synthetic AMPs with antibacterial properties and anti-inflammatory activity were created. Based on the analysis of the AMP structures, modified tryptophan-containing amphipathic helical undecapeptides (WALK11), exhibiting antimicrobial activity with significant anti-inflammatory potential, were synthesized [156]. With the use of cells of the mouse macrophage line RAW264.7, the WALK11 peptide was shown to inhibit the expression of inflammatory mediators IL-1β, IL-6, INF-β, and TNF-α, while maintaining antibacterial activity.

#### 3.1.3. AMPs’ Action on the Complement System

Alpha-defensin HNP-1 inhibits the classical and lectin pathways of activation of the complement system at an early stage, forming C1q and MBL complexes, thereby protecting the body from tissue damage [157].

Invertebrate AMPs can also affect the human complement system, and, depending on the concentration, the effect is multidirectional. In particular, the peptide arenicin-1 from the marine polychaete *Arenicola marina* at relatively low concentrations (1–40 µg/mL) stimulates complement activation and lysis of target erythrocytes, while at higher concentrations (80–160 µg/mL), arenicin acts as a complement inhibitor. The authors of this study discuss the possibility of interaction of AMPs with complement proteins, C1q and C3, and the regulation of their functional activity [158]. The influence of structural changes in arenicins on their interaction with complement proteins and biological activity was studied [159]. The arenicin-1 derivative without a disulfide bond (Ar-1- (C/A)), despite the absence of this bond, retains all important functional activities and also exhibits lower toxicity compared to the natural analogs previously discovered [160,161,162].

In another study, the AMP tachyplesin-1 from the horseshoe crab *Tachypleus trindentatus* complexed with the human C1q complement protein and triggered the classic complement pathway. The authors used this property of tachyplesin-1 to study the possibility of tachyplesin-1 to bind to the surface of human prostate carcinoma TSU cells, and to attract proteins of the complement system to destroy carcinoma. It was found that the cytotoxic effect of tachyplesin-1 and C1q on human prostate carcinoma cells TSU is realized only if the spatial structure of the peptide is preserved, since the reduction and alkylation of disulfide bonds of tachyplesin-1 led to weak binding to C1q and less cytotoxic effect [163].

Obviously, AMPs can be considered as promising compounds for creating new therapeutic agents that regulate the work of the complement system, both with the aim of destroying infected and transformed cells, and with the aim of preventing complement activation.

### 3.2. The Effect of AMPs on Immunocompetent Cells. Chemotactic Activity of AMPs

Along with direct inactivation of bacteria, fungi and viruses, AMPs exhibit different effects on the cells of a host organism. Interaction of AMPs with immunocompetent cells of the human and animal body with their further activation so as to form an adequate immune response to the pathogen is an important property of AMPs. Recently, it has become obvious that the main function of AMPs is not only to directly destroy the pathogen at the initial stage, but also to attract phagocytic and cytotoxic cells for the elimination of killed bacteria at a later stage, trigger inflammatory reactions in the case of ineffectiveness of the initial stage and induce anti-inflammatory reactions for relief, completion of the process of inflammation and restoration of damaged epithelium.

The attraction of immunocompetent cells to the inflammation zone is carried out through the expression of chemokines and their receptors. The AMP triggering of the proinflammatory immune response is carried out with the participation of both the humoral factors described above and the cooperative interaction of immunocompetent cells.

AMPs possess a chemotactic activity for neutrophils, macrophages, and immature dendritic cells [164,165] and cause mast cell degranulation [166,167]. Defensins attract neutrophils to the area of inflammation, as well as cells that express the human chemokine receptor CCR6. It has been established that human alfa-defensins caused monocyte chemotaxis in vitro. HNP-1 demonstrated the most significant activity, HNP-2 was less active, while HNP-3 did not display a chemotactic effect [165]. These AMPs also caused chemotaxis of immature human dendritic cells and naive T-lymphocytes [168]. Beta-defensins hBD1–3 also induced chemotaxis of T cells and immature dendritic cells by binding to the chemokine receptor CCR6 or CCR2 [169,170,171]. Furthermore, beta-defensins stimulated the migration of keratinocytes [152] and endothelial cells of the human umbilical cord [172].

Beta-defensin-induced chemotaxis was sensitive to the pertussis toxin and was inhibited by antibodies to CCR6 [169]. CCR6 is predominantly expressed by immature monocytic dendritic cells (DC) and CD8 + T cells [166,168,173]. As a result of cooperative interaction, maturation of DCs from monocytes occurs. DCs activate CD4 + T cells and CD8 + T cells, as well as B cells. At this point, defensins induce the release of proinflammatory cytokines IFN- γ, IL-6, and IL-10 from monocytes [107,174,175]. However, it has also been shown that beta-defensins can recruit CD4 + T cells and dendritic cells through another CCR6-independent, not yet identified, receptor [176].

In addition, alpha defensins induce the expression on CD4+ T-lymphocytes of the co-stimulatory molecules CD28, CD152\CTLA 4 and CD11a\LFA1 [177]. Under the action of beta-defensins, monocytes and Th17 produce the cytokines IL-17, IL-22, and TNF- α, which can increase inflammation, limiting the spread of the infectious process [178].

Investigation of the mechanism of the effector action of hBD-3 on T cells demonstrated that hBD-3 induced tyrosine phosphorylation of STAT1 and suppressed tyrosine phosphorylation of STAT1 in the case of IFN-γ exposure. Signaling pathways initiated by hBD-3 can lead to an increase in various T cell effector functions during T cell receptor activation, such as an increase in IL-2 and IL-10 levels. hBD-3 simultaneously initiated the signaling cascade of tyrosine kinase and tyrosine phosphatase, which can simultaneously activate T cells and inhibit their response to other immune mediators [179]. The immunosuppressive role of hBD-3 has been confirmed in vitro on human peripheral blood monocytes and in vivo on mouse macrophages hBD-3 and the mouse orthologue Defb14 (but not hBD-2) effectively inhibited the production of LPS-induced serum TNF-alpha and IL-6 [180].

hBD-2 and hBD-3 can regulate their own production as well as the development and function of Treg and Teff cells. Analysis of the expression of the specific marker of regulatory T cells (Tregs) FoxP3 when incubating T cells with hBD-2 and hBD-3 showed an increase in CD4+CD127-CD25+ Treg after 18 h and a decrease in Treg after 42 h of incubation in vitro due to loss of the FoxP3 expression. hBD-2 and hBD-3 control polarization of human CD4+ T cells and their ability to induce differentiation of effector T cells into RORγt + Tbet + (Th17/Th1) cells and Treg cell differentiation. This plasticity of the T cell phenotype also allows them to convert from Tregs to an effector T cell phenotype like Th1/17 after 18 h of culture. By 42 h of culture, treatment with hBD-2 and hBD-3 induced the differentiation of both Teff and Treg cells towards a Th17-like phenotype. Compared to hBD-2, hBD-3 caused a more pronounced effect of increasing RORγt levels in CD4+ T cells. This increased expression may be responsible for the induction of an increased IL-17A secretion. It was also found that hBD-3, but not hBD-2, was able to induce a higher level of the IL-17A secretion. In addition, treatment with hBD-3 induced an increased expression of IL-6, which was capable of directing the differentiation of naive T cells towards IL-17-producing Th17 cells. These data indicate that hBD-2 can inhibit the ability of Treg cells and cause suppression of Teff cell activity. Interestingly, co-cultivation with hBD-2 also increases the resistance of Teff cells to Treg immunoregulation in vitro. The use of genetic analysis on microarrays identified the chemokine ligand CC-motif 1 (CCL1) as a potential gene responding to the effects of hBD-2. It turned out that CCL1 blockade inhibited the suppressive function of Treg. The effect of hBD-2 and hBD-3 on Treg and Teff demonstrates the plasticity of T-cell phenotypes and the indirect effect of defensins on adaptive immunity [181].

Opposite data were obtained when hBD-2 was exposed to human peripheral blood T cells, in which stimulation of IFN-γ and IL-10 and suppression of IL-17 production were observed. Perhaps, the plasticity of T-lymphocytes and their dependence of the microenvironment and the duration of exposure to beta-defensins can serve as a presumable explanation [182].

The human alpha-defensins are chemoattractants for macrophages, T-lymphocytes, and mast cells [183]. In the analysis of cross-regulation between human alfa- and beta-defensins, it was found that the alfa-defensin receptor was cross-desensitized by beta-defensins. In contrast, alfa-defensins desensitize beta-defensin-mediated migration of immune cells, which indicates joint receptors for both defensin families [183].

While alpha- and beta-defensins stimulate the proliferation of T cells, another AMP, human cathelicidin LL-37, showed chemotactic activity for neutrophils, monocytes, and CD4+ T-lymphocytes [184] and induced apoptosis in regulatory T cells [185].

AMPs can limit inflammation. For example, depending on the microenvironment, alfa-defensins can block the secretion of IL-1β by monocytes activated by lipopolysaccharide (LPS) [186].

The effect of defensins on human monocytes depends on the maturity of the immune system. When investigating the effect of hBD-1 on neonatal umbilical monocytes, hBD-1 was found to induce the production of GM-CSF and IL-4, but not inflammatory cytokines. In this case, hBD-1 promotes the differentiation of neonatal monocytes from umbilical cord blood into immature dendritic cells (DCs) and then the final maturation of DCs. In addition, hBD-1 inhibited apoptosis through CCR6 in dendritic cells derived from neonatal monocytes. In relation to neonatal CD4+ T cells, hBD-1 promoted proliferation and activation, but not their maturation [187].

hBD-2 and hBD-3 activate plasmacytoid dendritic cells (pDCs), increasing intracellular uptake of CpG. In this case, CpG and host DNA form aggregates with hBD-2 and hBD-3 [145]. The effect of defensins on B cells is indirect, and it is realized through the interaction of B and T cells. This increases the systemic response and the synthesis of IgG, but not IgA, due to the assistance provided by the cytokines Th1 and Th2 [135].

Manifestation of multivarious effects of AMPs depends on their concentration in blood. At pico- or nanomolar concentrations, they can bind to certain receptors on the cell surface and cause, for example, chemotaxis of immunocompetent cells. At micromolar concentrations, which are observed in an infectious process and inflammation, AMPs exhibit antimicrobial activity and can have a toxic effect on the cells of the host organism.

In particular, the concentration of defensins is critical for the realization of their activity. At 5 μM and higher, hBD-3 can cause damage to monocyte membranes (but not membranes of B and T cells) due to interaction with negatively charged phospholipids [188]. Similarly, the concentration of HNPs released into the microenvironment upon activation of neutrophils during inflammation exerts a differential effect on cytokine production in activated monocytes. HNP concentrations from 1 to 10 nM can upregulate the expression of tumor necrosis factor alfa (TNF-α) and interleukin-1β (IL-1β), whereas concentrations from 10 to 100 µM are cytotoxic to monocytes [186]. The revealed selectivity of the activity of various human defensins indicates the presence of a fine regulation mechanism of immune homeostasis by AMPs [149].

AMP cathelecidin LL-37 attracts monocytes, neutrophils, and T-lymphocytes to the inflammation focus, interacting with the surface receptor FPRL1 (formyl peptide receptor-like 1) presented on these cells [184].

The wide spectrum of defensin activity on immunocompetent cells, their selectivity and ability to shift the proinflammatory response to an antiinflammatory one have led to the assumption that the immunomodulatory activity of defensins is no less important than the antibacterial activity, and it serves as a key factor in the binding of the innate and adaptive immune response [136,189,190].

When comparing the cytotoxic and antibacterial properties of alpha- and beta-defensins, it was found that beta-defensins may be more suitable antimicrobial agents for clinical use than alpha-defensins due to a less pronounced cytotoxic effect [151], and the most active among beta-defensins, hBD-3, can be considered as a candidate AMP [191,192].

The properties of other immune cells can also be altered by the action of AMPs, which can initiate degranulation of mast cells, participating in the development of inflammatory and allergic reactions. Mast cells are highly specialized cells playing a key role in the development of inflammation. When mast cells are activated, a broad spectrum of different molecules are released and act as mediators of inflammatory reactions. Both human α- and β-defensins have been shown to cause degranulation of mast cells [193,194,195]. Human cathelicidin LL-37 also exhibits this type of activity [194]. LL-37 induces the release of histamine by mast cells, as well as the secretion of IL-1β, IL-4, and IL-5 [167]. Thus, AMPs from neutrophils and barrier epithelium can be involved in the development of inflammation, chemotaxis and degranulation of mast cells.

AMPs can not only affect immunomodulating cells, they are also mediators of endocrine–immune interactions and have corticostatic activity. It has been shown that beta-defensins are expressed in human and mouse pancreatic endocrine cells [196], weaken the autoimmune response, and reduce the subsequent development of diabetes by increasing the proliferation of pancreatic beta cells and the number of Treg cells. It has been proven that changes in the AMP repertoire in tuberculosis are associated with the severity of the disease, the clinical picture, specific therapy, and the level of immunoendocrine mediators. In newly diagnosed patients with pulmonary (PTB) or pleural tuberculosis (PLTB), it was found that severe PTB patients displayed higher circulating amounts of hBD-3, statistically different from control ones. At the same time, LL-37 concentrations appeared within the normal range. PLTB patients revealed decreased levels of hBD-2 and increased amounts of hBD-3 and LL-37 in pleural fluids and plasma. Considering the immune-endocrine dysregulation in tuberculosis, there were detected positive correlations between levels of cortisol, IL-6 and β-defensin-3 in plasma from untreated severe patients and their dehydroepiandrosterone and LL-37 values. The different profile of PLTB patients, decreased hBD-2 along with increased hBD-3 and LL-37 levels, suggests a differential role of these hDPs in a host defense [197]. The discovered correlation raises the question of causation, the answer to which might be provided by further studies aiming to prove that dehydroepiandrosterone promotes the production of hBD-2 and hBD-3 in infected cells, correlating with the decrease of *Mycobacterium tuberculosis* bacilli loads [198].

Alfa-defensin inhibited ACTH-induced corticosterone production by rat adrenal cortex cells in vitro [199]. This defensin also inhibited ACTH-induced aldosterone synthesis by rat adrenal cells, but had no effect on angiotensin II-stimulated aldosterone production [199], although it inhibited aldosterone synthesis induced by α-melanocyte-stimulating hormone [200]. It was found that the administration of the RatNP-3 defensin immediately before stress exposure reduced the stress-induced increase in blood corticosterone concentration and normalized stress-induced changes in the number of neutrophilic granulocytes in the blood of rats [201].

There was no correlation between human defensins (HNP-1 and hBD-1) and cortisol and testosterone levels in a study of defensins and hormones levels in athletes experiencing prolonged physical activity. Observations during 12 months showed a 29% increase in HNP-1 levels after 3 months, and a 10-fold increase in hBD-1 after 6 months, which persisted throughout the entire observation period. At the same time, cortisol and testosterone levels peaked at 6 months, and returned to their original levels after 12 months [202].

In addition, beta-defensins are expressed in different segments of the testis [105], and have a main function in sperm maturation. A beta-defensin mutation at the *DEFB126* locus was found to decrease sperm motility and fertility in men [203]. Mice with deletion of two or more beta-defensin genes are infertile [204]. Alpha-defensin HNP-1 can restore the ability to proliferate Schwann cells, influencing the regeneration of peripheral nerve damage, inhibiting cell aging and apoptosis [205].

The effect of defensins on the intestinal mucosa is decisive for maintaining homeostasis. The absence of defensin expression contributes to an increase in the number of pathogenic bacteria and is observed in inflammatory bowel diseases [206]. At the same time, an increased amount of defensins may indicate the intensity of the immune response when the content of opportunistic pathogens is increased [207].

Currently, more and more data point to participation of AMPs in the interaction between the innate and acquired immunity systems. The effect of AMP on different types of immunocompetent cells may be direct or intermediated [208]. AMPs have an effect on the functional activity of dendritic cells, which, in turn, modulate the activity of lymphocytes. AMPs are also produced by the immunocompetent cells, which can secrete these molecules during the development of the immune response. As a result, special immunomodulatory activity of AMPs are realized [209].

## 4. Allergenic Activity of AMPs

AMPs, which evolved to protect a host against pathogens, perform a number of other functions in multicellular organisms, such as an influence on chemotaxis, cell differentiation, synthesis of chemokines, cytokines, corticosteroids, maturation of germ cells, and regeneration of tissues, including peripheral nerves. Furthermore, AMPs are also involved in the regulation of inflammation processes. When analyzing the effects of AMPs on humans, it should be noted that some AMPs possess allergenic properties. This should not be overlooked with consideration to prospects of their medical use.

It has been shown that some plant AMPs belonging to pathogenesis related (PR) proteins, induced by abiotic and biotic stress factors and found in roots, leaves, stems, pollen and fruits of plants, can cause allergic reactions in humans up to anaphylactic shock with a fatal outcome [210,211,212,213,214].

It was revealed that out of 19 classes of PR proteins, the classes 2, 3, 4, 5, 8, 10 and 14 demonstrated allergenicity. Moreover, structural homology was found between some groups, which explains the cross-reactivity [215,216,217,218,219]. The use of modern approaches to the determination of structural homology, including molecular docking, revealed that all 19 classes of known plant PR proteins including AMPs have a potential allergenic ability [220,221,222]. At the same time, the expression of endogenous AMPs in the same allergopathology can differ significantly. The skin, mucous membrane of the eyes, nasopharynx, digestive and reproductive systems, as well as the lungs perform barrier functions [223], and AMPs are the first line of defense against pathogens.

Allergic rhinitis (AR) is manifested in excessive secretion of mucus, mucosal edema and difficulty breathing. Analysis of AMP expression in the tonsils of patients with AR showed significantly lower levels of hBD2 compared to patients with non-allergic rhinitis [224]. The data correlate with the results of comparing the level of beta-defensin in the nasal fluid of children with AR in comparison with healthy ones. It was found that in children with AR, the levels of β-defensin 2 in the nasal fluid are reduced compared with the control group, and the levels of β-defensin 2 are negatively correlated with the severity of the disease: 173.8 pg/mL (interquartile range: 54.8– 205.9 pg/mL) in the allergic rhinitis group and 241.6 pg/mL (163.5–315.2 pg/mL) in the control group [225]. The levels of cathelicidin LL-37 in the nasal fluid of children with AR were also lower than in the healthy group (median 2.3 ng/mL (minimum–maximum, 2.1–3.2 ng/mL) versus 2.6 ng/mL (2.1–5.4 ng/mL). The severity of AR manifestation negatively correlated with the level of LL-37 in the nasal fluid. All these data emphasize the role of AMPs in the pathogenesis of AR [226]. Interestingly, according to the data of a proteomic study, other peptides and proteins are significantly less expressed in the nasal secretion in patients with AR in comparison with healthy people during the AR exacerbation season. Outside the season of flowering and pollen, in healthy people, the representation of peptides and proteins in the nasal secretion is significantly reduced, and in patients with AR, such a decrease is not found, and outside the season, their proteome/peptidome is superior to healthy people. These results indicate the absence of plasticity of the reaction of the nasal mucosa in patients with AR [227]. In non-allergic rhinosinusitis, exposure to fungal allergens from Aspergillus and Alternaria increased expression of cathelicidin LL-37 in nasal tissues in patients with chronic sinusitis by 4- and 6-fold (respectively) [228], which indicates differences in the pathogenesis and mechanisms of allergic and non-allergic sinusitis.

Very often, AR is accompanied by allergic conjunctivitis. In a study of AMP expression in the tonsils of patients with allergic rhinoconjunctivitis, it was shown that the level of hBD1–3 in patients with allergic rhinoconjunctivitis is significantly reduced compared to healthy donors [229]. Human corneal epithelial cells constitutively produce the antimicrobial peptides cathelicidin LL-37 and beta-defensins [230]; their expression can be increased by 3.75–4.9 times in vitro in response to heat-inactivated *Candida albicans* with peak values after 4 h of incubation [231]. Reduced levels of AMPs in patients with allergic rhinoconjunctivitis may be due to exposure to Th2 cytokines, which inhibit the production of AMPs [229]; in addition, the presence of genetic polymorphisms of the hBD genes is possible, as well as an influence of other factors on AMPs expression [232]. It was shown that the severity of manifestations of allergic diseases of the oral cavity (lichen planus and recurrent aphthous stomatitis, etc.) was largely associated with genetic polymorphisms of the hBD-1 genes [233,234,235,236]. hBD-1 gene polymorphisms are also associated with susceptibility to pulmonary infectious diseases, including asthma [237] and chronic obstructive pulmonary disease (COPD) [238]. In the analysis of the main polymorphism variants of the hBD-1 genes rs1047031 (C/T), rs1799946 (C/T), rs2738047 (C/T) and rs11362 (C/T) by genotyping 575 blood samples of men and women, cigarette smokers (288)/non-smokers (287), it was found that the CT and CT+TT rs1799946 genotypes showed a 5-fold increased correlation among smokers compared to control women, but with protective effects in the TT genotype in non-smokers [239]. In this regard, when analyzing the level of hBD expression in asthma, it is necessary to take into account the possibility of genetic polymorphisms of AMP genes, which can aggravate the course of asthma [240]. Asthma is also associated with polymorphisms not only in the hBD-1 gene, but also in the hBD-2 gene. Genetic changes at the hBD-2 gene locus or the absence of *DEFB4A* are strongly associated with the prevalence of asthma and allergic diseases in children. Moreover, the early administration of hBD-2 to animals in the asthma model prevented the development of asthma, thus, hBD-2 can play an important role in preventing severe forms of asthma [241]. These results are supported by another study on the role of hBD-2 in asthma development in a steroid-responsive house dust mite allergic respiratory disease model and in a steroid-insensitive ovalbumin model. In both models, therapeutic intranasal application of hBD2 significantly reduced the influx of inflammatory cells into bronchoalveolar lavage fluid and reduced allergic inflammation of the upper respiratory tract in mice. The results obtained may be of great practical importance for the treatment of allergic and especially steroid-resistant asthma [242].

Constitutive expression of hBD-1 in airway epithelial cells contributes to protection against bacterial and viral infection, while hBD-2, hBD-3, and hBD-4 are induced by various stimuli and are found in asthma [243,244,245]. High concentrations of alpha- and beta-defensins have a negative effect on pulmonary epitheliocytes, causing migration of neutrophils, inducing mucus hypersecretion, promoting degranulation of mast cells, and increasing vascular permeability [194,246].

Degranulation of mast cells under the action of alpha- and beta-defensins with the release of eicosanoids and the production of IL-31 increase vascular permeability, and in atopic dermatitis (AD) cause itching and damage to the skin. An excess of alpha- and beta-defensins has a negative effect on AD [247]. On the other hand, in AD there is also a lack of production of alpha- and beta-defensins, contributing to colonization by microbial organisms, mainly *Staphylococcus aureus* and *Herpes simplex* virus [248,249]. The mechanism of the protective action of AMPs includes strengthening the tight-junction barrier functions of keratinocytes through activation of the expression of surface receptors, blocking dendritic cell TLR4 activation and allergic contact sensitization [250,251].

Thus, evaluating the contribution of AMPs to allergic inflammation, we can conclude that an excessive amount of AMPs can provoke serious pathological changes. At the same time, a lack of production is accompanied by infectious complications on the skin and mucous membranes.

## 5. Conclusions

A wide spectrum of antimicrobial activities of AMPs inspires researchers to search for new active peptides in biological material [252,253], and also serves as the basis for the design of new synthetic compounds [254,255]. To date, information about AMPs is presented in numerous databases describing the structural, functional, allergenic and toxicological properties of these peptide factors of innate immunity [256,257,258,259,260]. The tools of modern bioinformatic approaches allow one to perform an in-depth analysis of structural homology in order to search for new, not yet discovered AMPs, and also offer additional methods for predicting a putative biological activity in silico [261,262,263,264]. A cytostatic activity of AMPs toward normal cells, as well as activities of proteases in bloodstream and interaction with host plasma proteins, are serious obstacles for the medical use of AMPs. In this connection, the tasks of searching for compounds that selectively affect the target, selection of doses, and the method of application become urgent.

AMPs are present in all organisms, from unicellular to multicellular, and have a wide spectrum of action. AMPs show a direct effect on neutrophils, monocytes, dendritic cells, T-lymphocytes and mast cells, initiating innate immunity. AMPs act on B-lymphocytes indirectly, enhancing the induction of antigen-specific immunity, which ultimately leads to the activation of adaptive immunity. The adjuvant activity of AMPs in relation to bacterial and viral antigens served as the basis for the inclusion of AMPs in vaccines and made it possible to formulate the concept of a “defensin vaccine” as a conceptual basis for development of novel vaccines.

Thus, immunomodulatory functions of AMPs include an influence on cells in the nearest microenvironment, recruitment and activation of other cells, supporting the response to pathogenic microorganisms and completing the inflammatory process, thus exhibiting a systemic effect.

**Figure 2:**
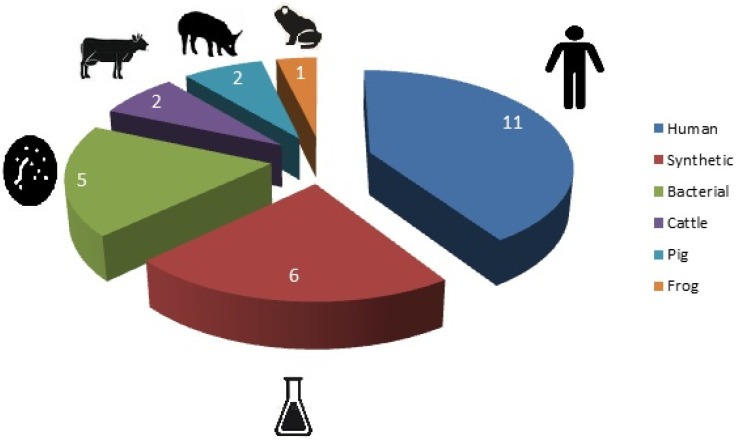
Sources of AMPs under clinical trials.

AMP research is of fundamental and applied importance. On the one hand, AMPs are a model for studying immunity and protection of various organisms against infections, analyzing the evolution of protection modes, as well as the etiology of autoimmune and allergic diseases. On the other hand, a large number of molecules with antimicrobial properties can be the basis for the development of drugs, which are especially important under the present-day conditions of the emergence of resistance in microorganisms to antibiotics. Currently, clinical trials of recombinant and synthetic AMPs and those derived from natural sources are underway (Figure 2) [265].

Thus, the initially detected antimicrobial activity of AMPs in humans is not the only one, and, possibly, their immunomodulatory activity makes an equal contribution to the manifestation of effector functions. For the successful use of AMPs in medical practice, it is necessary to study in detail their immunomodulatory activity, taking into account their pleiotropy, the degree of maturity of the immune system and the microenvironment in order to prevent complications and increase the effectiveness of therapy. It should also be taken into account that the real functions of one or another AMP depend on the type of total regulatory effect on the target cell, and not only on the individual properties of the given peptide.
